# Application of 3D printing and framework internal fixation technology for high complex rib fractures

**DOI:** 10.1186/s13019-020-01377-8

**Published:** 2021-02-15

**Authors:** Xuetao Zhou, Dongsheng Zhang, Zexin Xie, Yang Yang, Menghui Chen, Zheng Liang, Guoliang Zhang, Shujun Li

**Affiliations:** 1 Department of Thoracic Surgery, the Second Hospital of Hebei Medical University, No. 215, Heping west road, Shijiazhuang, 050000 China; 2 Department of Cardiothoracic Surgery, Shijiazhuang No. 3 Hospital, Shijiazhuang, 050011 China

**Keywords:** High complex rib fractures, 3D printing, Framework internal fixation, Preoperative plan

## Abstract

**Objective:**

To explore the clinical effect of 3D printing combined with framework internal fixation technology on the minimally invasive internal fixation of high complex rib fractures.

**Methods:**

Total 16 patients with high complex rib fractures were included in the study. Before the procedure, the 3D rib model was reconstructed based on the thin-layer chest CT scan. According to the 3D model, the rib locking plate was pre-shaped, and the preoperative planning were made including the direction of the locking plate, the location of each nail hole and the length of the screw. During the operation, the locking plate was inserted from the sternum to the outermost fracture lines of ribs with screws at both ends. In addition, the locking plate was used as the frame to sequentially reduce the middle fracture segment and fix with screws or steel wires. Chest x-rays or chest CT scans after surgery were used to assess the ribs recovery. All patients were routinely given non-steroidal anti-inflammatory drugs (NSAIDS) for analgesia, and the pain level was evaluated using numerical rating scale (NRS).

**Results:**

The preoperative planning according to the 3D printed rib model was accurate. The reduction and fixation of each fracture segment were successfully completed through the framework internal fixation technology. No cases of surgical death, and postoperative chest pain was significantly alleviated. Five to 10 months follow up demonstrated neither loosening of screws, nor displacement of fixtures among patients. The lungs of each patients were clear and in good shape.

**Conclusion:**

The application of 3D printing combined with framework internal fixation technology to the high complex rib fractures is beneficial for restoring the inherent shape of the thoracic cage, which can realize the accurate and individualized treatment as well as reduces the operation difficulty.

## Background

Chest injuries often cause multiple rib fractures and serious complications [1]. At present, the surgical fixation of multiple rib fractures has been widely used in the world, which can achieve good results [2]. Besides, with the development of internal fixation equipment and the continuous in-depth research, surgical methods have become minimally invasive. However, surgery of high complex rib fractures is still considered difficult due to the position of fractures, the coverage of strong pectoral muscles (mammary gland in women) the complex structure of anatomy and multiple fracture segments. The rib fractures meet the following requirements are classified as high complex rib fractures: (1) has one to three fractures in the 2nd to 4th ribs; (2) each fracture site has ≥3 fracture segment; (3) the length of the middle fracture segment≤5 cm; (4) the presence of costal cartilage fracture. At present, fractures involving this part are usually widely separated from the chest wall muscle layer by a diagonal incision of the chest muscle or subbreast fold, resulting in long incisions and large injuries [3]. Minimally invasive tunnel fixation with minimally invasive plate osteosynthesis (MIPO) has a tiny surgical incision, whereas the visual field is not well exposed [4]. In addition, the locking plate shaping during the procedure is often difficult, time consuming and requires multiple adjustments due to the multi-segment fractures of ribs accompanied with significant deformities of chest curvature, longitudinal torsion rate and deployment curvature [5]. Furthermore, locking plate is difficult to achieve a good fit with the ribs, resulting in increased probability of thoracic deformity, and displacement of fixator and screws after surgery. Some studies have found that insufficient fixation can lead to fracture nonunion, delayed union and secondary surgical fixation [6]. Therefore, preoperative planning and intraoperative reduction of rib fracture are the key factors to achieve good clinical outcomes in internal fixation of high complex rib fractures.

Computer tomography (CT), three-dimensional (3D) reconstruction, and 3D model printing technology have been successfully used in the preoperative planning of a variety complex cases such as fractures and reconstruction of chest wall defects [7–9]. There are also reports showing that 3D printing is beneficial for internal fixation of rib fractures [10, 11]. However, not many studies have demonstrated the use of 3D printing technology for preoperative planning, and the specific operation skills of high complex rib fractures. Therefore, this study aimed to explore the feasibility and clinical efficacy of pre-shaping locking plates based on 3D printed rib models, as well as the application of framework internal fixation techniques in minimally invasive surgery of high complex rib fractures.

## Methods

### Participant data

Sixteen subjects who underwent surgical treatment of high complex rib fractures in the Shijiazhuang third hospital from June 2016 to August 2019 were included in this study. The inclusion criteria of participants were as follows: (1) had ≥3 rib fractures, with obvious misalignment (more than half of the rib width showed by CT horizontal axis images); (2) had at least one to three fracture sites from the 2nd to 4th ribs, each≥3 fracture segment and accompanied by costal cartilage fracture; (3) the length of the middle fracture segment was ≤5 cm; (4) at least ≥6 points in the preoperative numerical rating scale (NRS) scores; (5) had 3D printing of rib fractures before surgery; (6) used framework locking plate internal fixation method during the operation. Exclusion criteria were as follows: (1) had no rib fractures (2nd to 4th); (2) did not use 3D printing technology for 2nd to 4th rib fractures; (3) did not use framework internal fixation technique during the operation. There were 12 males and 4 females participated in this study. The age ranged from 26 to 67 years old with the average of 50 years old. The causes of fractures including 12 cases of traffic accidents, 3 cases of fall injuries and 1 case of machine crush injury. There were 11 cases had unilateral complex rib fractures (2nd and 4th), 4 cases involved in unilateral complex fractures of 3rd and 4th ribs, and 1 case of the 2nd and 3rd complex rib fractures. The high complex rib fracture was diagnosed using a 64-row spiral computed tomography (CT) (GE company). This study was approved by the Medical Ethics Committee of Shijiazhuang third hospital (2018–010), and all patients have signed informed consents.

### 3D printing and preoperative planning

**Fig. 1:**
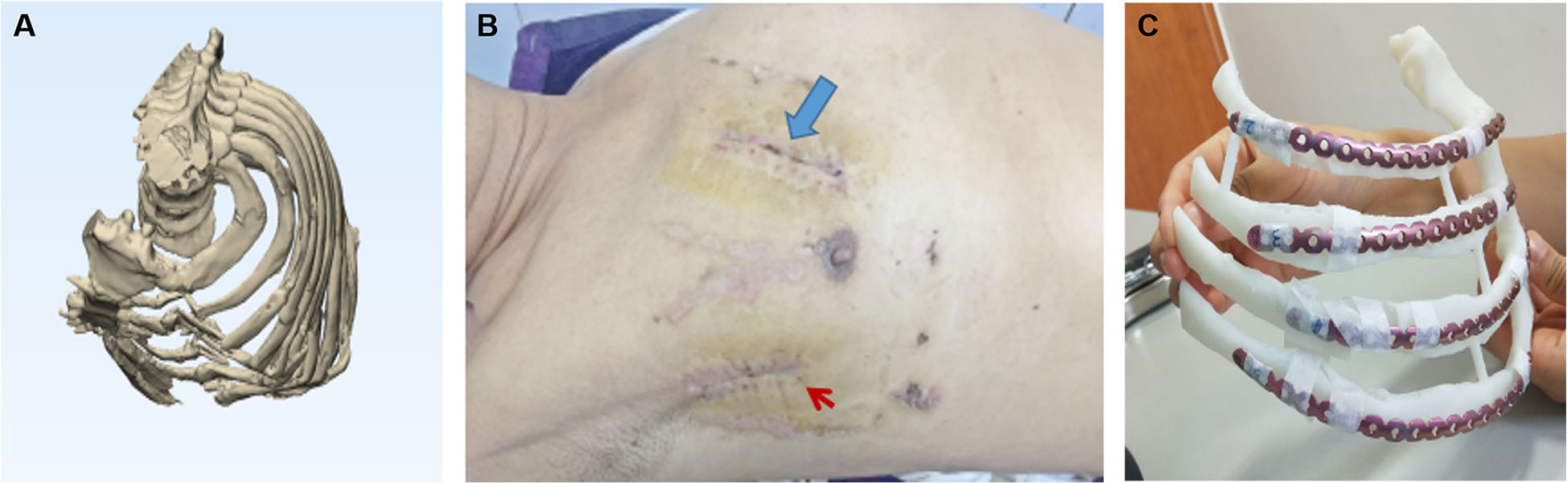
Preoperative planning. **a** 3D image of high complex rib fractures. **b** two small incisions approaches on the chest (shown by thick arrows) and underarms (shown as small arrows). **c** shaping the locking plate according to the 3D model

**Fig. 2:**
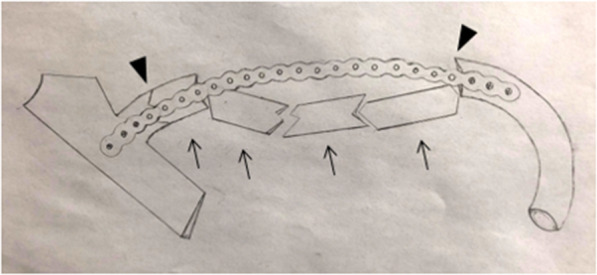
Schematic diagram of the framework fixation of the second rib. The two ends of the locking plate were fixed to the sternum and ribs outside the fracture line of the two ends to establish the “frame” structure, and each middle fracture section was lifted, reduced, and fixed to the “frame” in sequence. “▼“indicated fracture lines at both ends, “↑“indicated 4 fracture segments in the middle

**Fig. 3:**
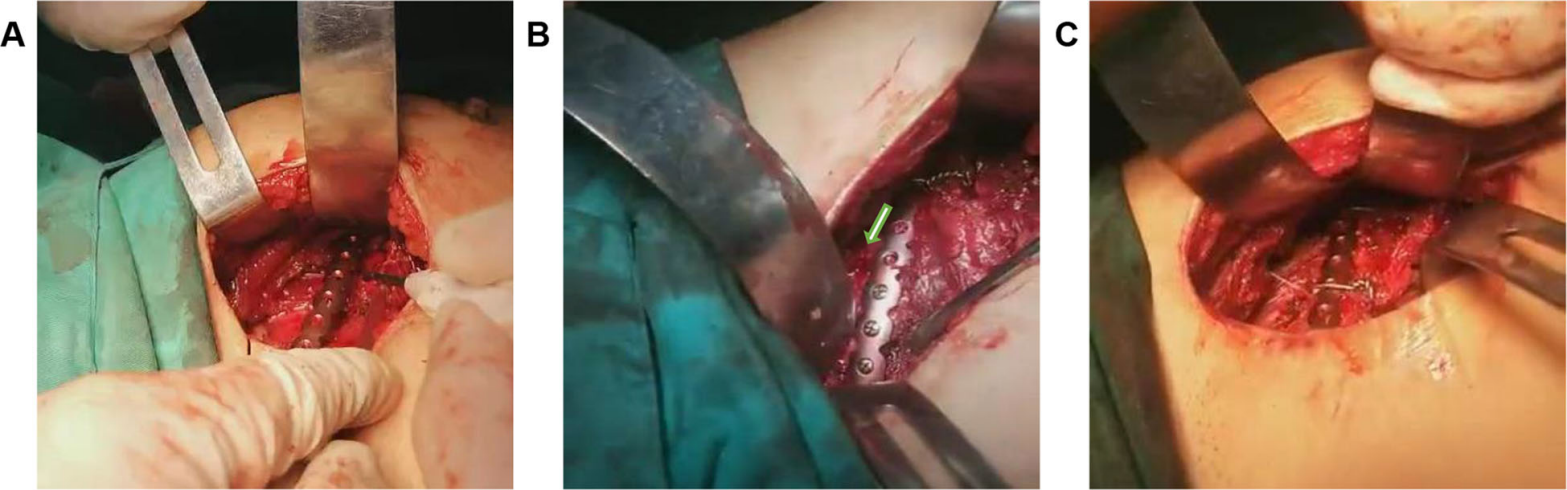
Operation diagram of framework locking plate internal fixation. **a** The shaped locking plate was inserted into the patient’s third rib through the right axillary incision. As expected before the operation, the two ends were well attached to the intended fixed position of the fracture line. There were multiple fractures in the middle, and the locking plate could easily travel through the space between the locking plate and the rib. **b** Through the right axillary incision, the patient’s third rib was fixed to the normal ribs at the distal end of the fracture line with 3 screws (arrows indicate the fracture ends). **c** The middle fracture segments were fixed to the locking plate with steel wires and screws. Deformities such as sunken ribs were corrected, and the shape recovered well

**Fig. 4:**
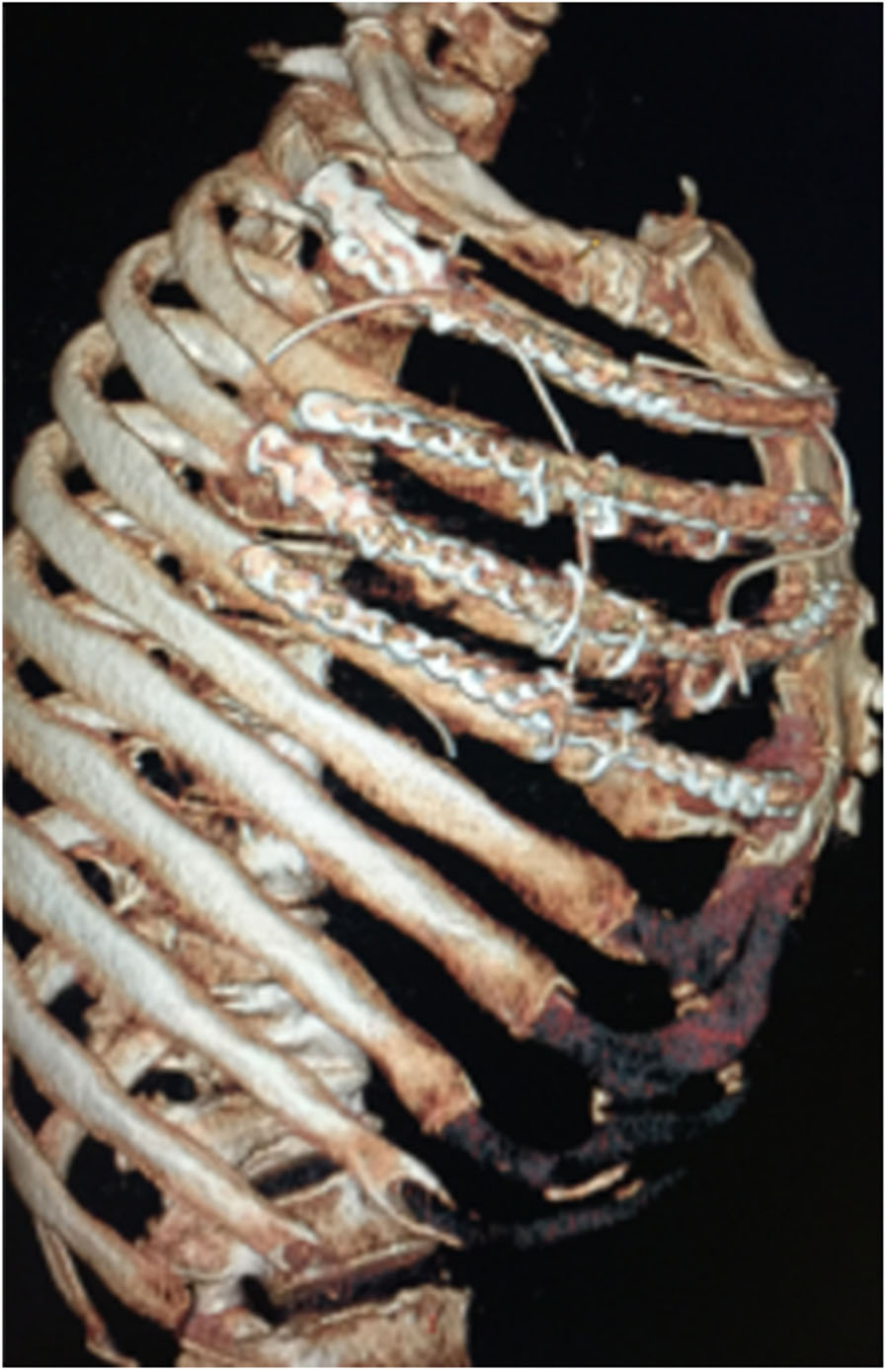
The locking plate reviewed by rib CT after surgery was consistent with that in preoperative plan

The results of multi-slices spiral CT scan were processed with the Medical image processing software (MDT2AB-010A, Meditool Medical Technology (Shanghai) Co., Ltd.) one or two days before surgery. Then, the processed data was imported into the 3D printer (pangu4.1, Meditool Medical Technology (Shanghai) Co., Ltd.) to make a true size photosensitive resin simulation model of the ribs. Before the surgery, preoperative planning was carried out in greater details as follows: (1) the morphological observation of 3D reconstruction model and roadmap including the characteristics and morphology of fractures, especially the small and free fracture fragments (Fig. 1a); (2) the selection of surgical incision site (Fig. 1b); (3) reducing 3D model fracture to its pre-injury form and shaping the locking plate (MtrixRIB, Synthes) according to the restored rib model (Fig. 1c); (4) record the placement position and the direction of the locking plate, and the length and number of screws; (5) Sterilization of the locking plate prior to use in the procedure.

### Framework locking plate internal fixation

All patients were placed supine on the table with the injection of general anesthesia and prophylactic intravenous antibiotics. The affected fracture side was elevated at 30 degrees. The upper limbs of the affected side were sterilized and wrapped with a sterile sheet with the forearm placed on the side of the body. Axillary and parasternal incisions corresponding to the fractured ribs were performed. Firstly, the muscular layer was separated layer by layer through the axillary incision, and the anterior serratus muscle was revealed through the outer edge of the pectoral muscle. According to the muscle-sparing principle, the surface of the ribs was reached through the muscle fibers and serratus anterior, avoiding transverse muscle fibers. The space between the pectoral muscles and ribs was opened along the surface of the ribs and fully freed to the end of the sternum. Once the second rib is involved in the procedure, the position of the upper limb on the affected side could be adjusted to relax the surrounding skin and muscles, helping to increase the visual field. Then, the parasternal incision was made to reveal the muscle layers, the fractured ribs and its corresponding sternal bone surface through the sternum muscle based on the muscle-sparing principle. A tunnel-like operating space was established between the axillary and parasternal incisions through the rib surface. The operation was fixed in order from head to foot side by side. According to the preoperative planning and marking, the shaped locking plate was inserted directly. The front part was fixed to the sternum with at least two screws, and the side was fixed to the ribs beyond the outermost fracture line with at least two screws to build “frame” structure, and then each fracture segment in the middle was lifted, reduced, and fixed to the “frame” in sequence (Figs. 2 and 3). According to the size and shape of the middle fracture section, either drilling, screw fixation or 0.6 mm steel wire strapping was adopted. If other fractured ribs are involved in the procedure, the incision sites need to be appropriately adjusted or selected according to the fracture. Thoracoscope examination was selected according to the lung contusion and pleural effusion. After the lungs were fully recruited, the operation was completed. The affected side was routinely placed with a 28F closed thoracic drainage tube connected to a water-sealed bottle, and a 14F drainage tube was placed on the surgical wound, and the incision was sutured in layers.

### Postoperative care

After the surgery, all patients were given non-steroidal anti-inflammatory drugs (NSAIDS) for analgesia, and the pain level was evaluated according to the NRS at 10:00 a.m. every day. Once the NRS score reaches 4 points or less, the analgesic drugs will be stopped. Chest radiographs or chest CT scans were performed on the second and seventh days after surgery. Follow-up were conducted at 5 to 10 months post-operation to assess the recovery.

## Results

A true size rib model printed by 3D printing technology using the patient’s CT scan had a strong three-dimensional sense, which helped to observe the fracture segment from different angles. In addition, the shaped locking plate was in a suitable length, and fitted well in 3D printing model. The locking plate was then placed and fixed smoothly during the operation without adjustment, resulting in a successful established “frame” structure. The middle fracture segment was also smoothly reset in sequence. After surgery, chest radiograph or chest CT showed that the locking plate was fixed in a satisfactory way and the morphology of chest wall was well recovered (Fig. 4).

**Fig. 5:**
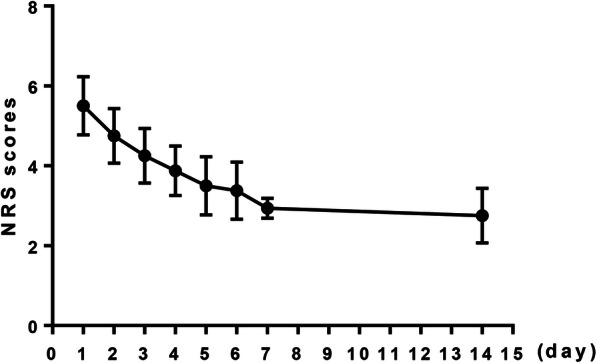
The NRS scores of the patients after surgery

All operations were performed by the same thoracic surgeon with the general situations showing in Table 1. Owing to the fixation of rib fractures in other parts or the exploration of the chest cavity had a great influence on the operation time, the fixation time of each rib fracture was recorded to describe the time accurately. After surgery, the intubation tube was removed successfully in all patients, and then returned to the general ward. As shown in Fig. 5, the chest pain of patients was relieved more than before surgery, and the NRS score gradually decreased with time. At the 7 days after the procedure, 14 patients had an NRS score of 4 or less, which means the analgesic drugs were stopped, however, NSAIDS was used again for two patients who had worsen chest pain after stopping the drug. Among these two patients, one stopped analgesic drugs 2 weeks after surgery, whereas another patient did not stop the analgesic drugs until 6 weeks after surgery. During the follow-up period, all patients recovered with well-shaped internal fixator, beautiful thoracic cage and healed rib fractures. No displacement or shedding of the fixator were demonstrated.

**Table 1:** General situation of rib surgery in 16 patients

Index	Mean (minimum-maximum)
Total operation time, min	120.0 (95–150)
Fixation time of each rib fracture, min	
Second rib	35.7 (33–40)
Third rib	33.3 (30–38)
Fourth rib	31.9 (31–36)
Surgery bleeding volume, ml	74.3 (50–100)
Parasternal incision length, cm	5.3 (4–6)
Axillary incision length, cm	7.2 (6–8)

## Discussion

In this study, the 3D printing and framework fixation of the other fractured ribs were not involved since the approaches of the internal fixation in that region were relatively simple, which had little help on the surgical process. In addition, the increase in 3D printing led to increased medical costs, which would increase the financial burden to patients. High complex rib fractures mainly occur in 2nd to 4th ribs, and often have strong chest muscles coverage in front and multiple fracture segments, making it difficult to reveal visual field and shape locking plate according to the surgeon’s experience. Conventional locking plate fixation requires the incision and the exposure length to be as close as possible to the length of the locking plate, which is not suitable for high complex rib fractures. Therefore, it is of significant to explore minimally invasive surgical methods and adjust the shaping locking plate for high complex rib fractures.

3D printing technology has been widely used in the medical field. Studies have shown that, it is easier to understand the three-dimensional shape of complex fractures with the help of 3D printing models, which is beneficial for preoperative planning, and makes the operation safer and more effective [12, 13]. In this study, the results showed that the length and shape of the locking plate shaped by 3D printing technology fitted well within the ribs and sternum, and the position was accurate without intraoperative adjustment. It saved the steps of intraoperative fracture reduction, as well as visual and empirical repeated shaping of the locking plate that were routinely used at this stage, thereby reducing tissue damage and shortening the operation time. In addition, two small incisions in the chest and underarms were performed in the surgery, instead of the incision through the chest muscles or under breast folds which is currently commonly used [3], which made the surgical incision greatly reduced and more beautiful. Under the full protection of muscles and neurovasculature, the “frame” structure was successfully established with completed reduction and fixation of each middle fracture section, allowing to reduce the steps of routine surgery on rib fractures and insert and temporarily fix the locking plates. The application of 3D printing and internal fixation technology reduced the operation time and difficulty of the procedure, which were consistent with other studies [11]. Therefore, the combination of 3D printing technology and framework fixation suggested the well-fixed rib fractures, well-fitted locking plate, and decreased probability of postoperative dislocation and screw shedding.

Chest wall pain is a strong and unpleasant subjective sensation in patients with rib fractures, which is related to the dislocation stimulation of rib fractures, release of pain factors, and compression of the intercostal nerves. Studies have shown that controlling of chest pain can not only reduce the patient’s own discomfort, but also prevent various complications such as pneumonia and respiratory limitation [14]. After surgical treatment, the fractured end of the ribs was fixed to avoid the movement of dislocation, and thus relieving the stimulation of the intercostal nerves caused by the fracture. This can significantly reduce the patient’s chest pain, and improve the quality of their life [15, 16]. This study also showed that most patients stopped using analgesic drugs 7 days after surgery, and the patients with chest pain were gradually relieved with time after surgery, indicating a satisfactory analgesic effect of 3D printing combined with framework internal fixation technology.

In summary, pre-shaping locking plate and preoperative planning of high complex rib fractures using the 3D printed rib model and framework fixation technology were feasible and effective in the minimally invasive surgery. This finding has not only solved the difficulty and inaccuracy of shaping locking plate during the surgery, but also simplified the surgical procedures and restored the shape of the thoracic cage perfectly. All patients had significantly reduced the chest pain after the procedure. The limitation of this study is the small sample size, which made it difficult to conduct randomized controlled trials to improve the reliability of the conclusion. Therefore, it is needed to continuously accumulate patient data in the clinic setting to increase the sample size for further research.

## Conclusion

The application of 3D printing combined with framework internal fixation technology to the high complex rib fractures is beneficial for restoring the inherent shape of the thoracic cage, which can realize the accurate and individualized treatment as well as reduces the operation difficulty.

## Data Availability

The datasets used and/or analyzed during the current study are available from the corresponding author on reasonable request.

## Abbreviations

NSAIDS: Non-steroidal anti-inflammatory drugs
NRS: Numerical rating scale
MIPO: Minimally invasive plate osteosynthesis
CT: Computer tomography
3D: Three-dimensional
